# Isolation and characterization of cultured chicken oviduct epithelial cells and *in vitro* validation of constructed ovalbumin promoter in these cells

**DOI:** 10.5713/ab.20.0627

**Published:** 2020-12-11

**Authors:** Hyeon Yang, Bo Ram Lee, Hwi-Cheul Lee, Sun Keun Jung, Ji-Youn Kim, Jingu No, Sureshkumar Shanmugam, Yong Jin Jo, Haesun Lee, Seongsoo Hwang, Sung June Byun

**Affiliations:** 1 Animal Biotechnology Division, National Institute of Animal Science, Rural Development Administration, Wanju 55365, Korea

**Keywords:** Chicken, Oviduct Epithelial Cells, Ovalbumin Promoter, Luciferase

## Abstract

**Objective:**

Transgenic hens hold a great promise to produce various valuable proteins. Through virus transduction into stage X embryo, the transgene expression under the control of constructed chicken ovalbumin promoters has been successfully achieved. However, a validation system that can evaluate differently developed ovalbumin promoters in *in vitro*, remains to be developed.

**Methods:**

In the present study, chicken oviduct epithelial cells (cOECs) were isolated from oviduct tissue and shortly cultured with keratinocyte complete medium supplemented with chicken serum. The isolated cells were characterized with immunofluorescence, western blot, and flow cytometry using oviduct-specific marker. Chicken mutated ovalbumin promoter (Mut-4.4-kb-pOV) was validated in these cells using luciferase reporter analysis.

**Results:**

The isolated cOECs revealed that the oviduct-specific marker, ovalbumin protein, was clearly detected by immunofluorescence, western blot, and flow cytometry analysis revealed that approximately 79.40% of the cells contained this protein. Also, luciferase reporter analysis showed that the constructed Mut-4.4-kb-pOV exhibited 7.1-fold (p<0.001) higher activity in the cOECs.

**Conclusion:**

Collectively, these results demonstrate the efficient isolation and characterization of cOECs and validate the activity of the constructed ovalbumin promoter in the cultured cOECs. The *in vitro* validation of the recombinant promoter activity in cOECs can facilitate the production of efficient transgenic chickens for potential use as bioreactors.

## INTRODUCTION

Transgenic hens have historically been considered ideal bioreactors due to several advantages, such as their short generation interval, low maintenance cost, and stability of recombinant proteins, compared with other species and provided a valuable tool for the production of therapeutic proteins in eggs [1]. To date, substantial progress has been made toward the generation of transgenic chickens and genetic modifications, including precise genome editing by virus transduction into Eyal-giladi and Kochav stage X embryos [2,3]. Recent studies have successfully produced recombinant proteins such as human epidermal growth factor [4], human lysozyme [5], and human neutrophil defensin 4 [6], which indicates that virus-mediated avian transgenesis could be useful for the establishment of bioreactors.

A chicken annually produces more than 300 eggs through its oviduct and chicken oviduct epithelial cells (cOECs) have unique features that are advantageous for the production of egg white protein, including ovalbumin, conalbumin, ovomucoid, and lysozyme. The chicken ovalbumin gene promoter, which leads to highly potent and tissue-specific ovalbumin expression, has been used for the production of transgenic hens [7]. In addition, ovalbumin promoters of different sizes, ranging from approximately 1.35-to-4.4-kb including the transcription or translation start sites, and estrogen response elements (EREs) have been developed in transgenic chicken and quail [8–10]. However, many limitations, such as absence of validation system that can evaluate expression and regulation of recombinant proteins driven by different ovalbumin promoters in *in vitro*, remain to be overcome.

Substantial efforts have been made to establish an *in vitro* system for the culture of cOECs for practical applications in avian transgenesis [11] and to understand the physiological and endocrinological roles of oviduct epithelium [12–15]. However, a simple isolation, culture, and characterization method for cOECs still remains to be challenging primarily due to relatively speedy growth and proliferation levels of other cells, such as oviductal tissue fibroblast cells. Therefore, an optimized establishment of cOECs is required to validate the transgene expression driven by a constructed ovalbumin promoter or evaluate variously developed oviduct specific promoters in *in vitro* prior to producing transgenic chickens.

In the current study, we developed a simple method for the isolation and characterization of cOECs and their subsequent cultivation to validate the constructed ovalbumin promoter through luciferase reporter analysis. This study will thus facilitate the optimization of recombinant protein expression in eggs through avian transgenesis.

## MATERIALS AND METHODS

### Experimental animals and animal care

The experimental protocol used in this study was based on an approved animal-use document and was in accordance to the guidelines of the Institute Animal Care and Use Committee (IACUC) of the National Institute of Animal Science (Approval No. 2017–219) at the Republic of Korea.

### Isolation and culture of chicken oviduct epithelial cells

To isolate cOECs, an oviduct tissue from an egg-laying White Leghorn (WL) hen (30 to 35 weeks) was dissected, separated and dissociated, and the method used for the isolation of oviduct cells was modified from that used in a previous study [14]. In this study, 15-to-20 cm-long oviduct tissue from the infundibulum to the magnum was separated from the oviduct, and the isolated tissue was washed with 70% ethanol, washed twice with phosphate-buffered saline (PBS; Gibco, Carlsbad, CA, USA) supplemented with 1% antibiotic/antimycotic (anti-anti; Gibco, USA), and placed on the petri dish. The mesosalpinx of the tissue was trimmed, gently torn off using microsurgical scissors horizontally to expose the inner surface, and soaked with fresh PBS to avoid drying. The inner surface was then delicately scraped several times with surgical blade, and the scraped tissue fragments were finely minced into approximately 2-mm fragments with microsurgical scissors. The minced tissue fragments were centrifuged at 700 g and 4°C for 10 min, and the supernatant was removed. The pellets were digested with 10 mL (1 mg/mL) of collagenase P (Roche, Basel, Switzerland) supplemented with 10 mM HEPES (Sigma, Saint Louis, MO, USA) and placed in an incubator with 5% CO_2_ at 37°C for 30 min, with vortexing every 5 min. The digested samples were centrifuged (1-step) at 700 g and 4°C for 10 min, and the supernatant was transferred with a fresh tube and centrifuged (2-step) again. The pellets from the first and second steps of the centrifugation were suspended in cell culture medium, and suspended tissue masses were totally placed on 100-mm collagen-treated dishes (Corning, New York, NY, USA) with Keratinocyte complete medium (K-SFM; Gibco, USA) supplemented with 5% chicken serum (Gibco, USA) and 1% anti-anti. The oviduct cells at passage 0 were isolated from the tissue masses in few days, observed under a microscope (Eclipse Ti, TE300; Nikon, Tokyo, Japan). Then, the cells from passage 1 were continuously cultured in K-SFM with 5% chicken serum, 1% anti-anti, and sub-cultured by trypsin treatment and centrifugation. The chicken embryonic fibroblast cells line DF-1 (CRL-12203; American Type Culture Collection, Manassas, VA, USA) were grown in Dulbecco’s modified Eagle’s medium (DMEM; Gibco, USA) supplemented with 10% fetal bovine serum (FBS; Gibco, USA), 1% anti-anti, and 2% chicken serum.

### Scanning and electron microscopy

The magnum portion of a chicken oviduct tissue was primarily fixed with 4% paraformaldehyde at 4°C, and washed three times with 0.05 M sodium cacodylate buffer. The sample was then secondarily fixed for 1.5 h with 1% osmium tetroxide in sodium cacodylate buffer, subjected to two brief washes, and stained overnight with 0.5% uranyl acetate at 4°C. For scanning and electron microscopy (SEM) analysis, the sample was dried twice with 100% isoamyl acetate for 15 min in a critical point dryer, mounted on metal stubs, coated with gold, and observed under a Bio-LV SEM (SN-3000; Hitachi, Tokyo, Japan).

### Chromosome karyotyping

The cOECs at passage 2 in a collagen-treated 75T flask were treated 200 μL of colcemid (Gibco, USA) stock solution, incubated at 37°C with 5% CO_2_ for 4 h and harvested by centrifugation at 1,000 rpm for 10 min. After aspiration of the supernatant, the cells were suspended in 5 mL of hypotonic solution (0.075 M KCl) and incubated at 37°C for 10 min. Subsequently, 500 μL of Carnoy’s fixative (ethanol: acetic acid = 3:1) was added to the cells, and the cells were again harvested by centrifugation as described above. After aspiration, 3 mL of Carnoy’s fixative was added to cells, and the cells were incubated for at least 20 min and harvested by centrifugation at 1,000 rpm for 10 min, these steps were repeated. The pellets obtained after the final centrifugation were spread on a glass slide, and the slide was baked at 60°C for 30 min, and the chicken chromosomes were stained with Giemsa.

### Reverse transcription polymerase chain reaction

RNA from the oviduct magnum tissue (positive control), DF-1 (negative control), and cOECs at passage 2 was extracted using an RNA mini preparation kit (Qiagen, Hilden, Germany). For reverse transcription polymerase chain reaction (RT-PCR) analysis, total RNA (0.5 μg) was used for cDNA synthesis with the Superscript IV First-Strand Synthesis System (Invitrogen, Carlsbad, CA, USA). RT-PCR was performed with HS prime Taq DNA polymerase (Genet Bio, Daejeon, Korea) using SimpliAmp PCR system (Applied Biosystems, Foster City, CA, USA). The primer sequences (Table 1) were selected from the reference or designed with NCBI Primer Blast. Primer pairs were used to amplify specific region into chicken oviduct ovalbumin, ovomucoid, estrogen receptor 1 (ESR1), occludin, cytokeratin 14, E-cadherin, and β-actin genes. The PCR conditions consisted of 94°C for 10 min, followed by 35 cycles of 94°C for 30 s, 58°C for 30 s, 72°C for 30 s, and 72°C for 5 min.

### Immunofluorescence

cOECs at passage 1 and DF-1 were seeded into a 4-well cell culture dish, fixed with 4% paraformaldehyde (Molecular probes, Eugene, OR, USA) and permeabilized with 0.5% Triton-X (Gibco, USA) for 15 min at room temperature. The cells were blocked with 5% bovine serum albumin (Molecular probes, USA) for 1 h at room temperature, primarily incubated with the rabbit polyclonal anti-ovalbumin antibody (1:250 [1 mg/mL] dilution, Abcam, Cambridge, UK) overnight at 4°C and then secondarily incubated with mouse anti-rabbit fluorescein isothiocyanate (FITC)-conjugated antibody (1:500 [2 mg/mL] dilution, Abcam, UK) for 1 h, and washed with PBS. Immunofluorescence were analyzed under a fluorescence microscope (DMI 6000 B; Leica, Wetzlar, Germany).

### Western blot

Protein from the oviduct magnum tissue (positive control), chicken leg muscle tissue (negative control), DF-1 (negative control), and cOECs at passage 2 were extracted using RIPA lysis buffer (Thermo Scientific, Waltham, MA, USA) supplemented with a protease and phosphatase inhibitor cocktail (Thermo Scientific, USA). The protein concentration was determined with the Bradford assay (Bio-Rad, Hercules, CA, USA). Protein extracts (15 μg) was electrophoresed with a sodium dodecyl sulfate-polyacrylamide gel electrophoresis 4% to 12% gel system (Invitrogen, USA) and transferred to polyvinylidene fluoride (Invitrogen, USA) membrane. The membrane was blocked with 5% skim milk in PBS (Thermo Scientific, USA) for 1 h and then primarily incubated with rabbit polyclonal anti-ovalbumin antibody (1:1,000 [1 mg/mL] dilution, Abcam, UK) and rabbit monoclonal anti-vinculin antibody (1:1,000 [0.054 mg/mL] dilution, Abcam, UK) overnight 4°C. The membrane was washed and then secondarily incubated with mouse anti-rabbit HRP conjugated antibody (1:2,000 [0.4 mg/mL] dilution, Santa Cruz, Dallas, TX, USA) for 30 min at room temperature. Amersham ECL prime (GE Healthcare, Buckinghamshire, UK) substrate was used to visualize the target bands, and the bands were analyzed using EZ-Capture II (Atto, Tokyo, Japan).

### Flow cytometry

cOECs at passage 2 and DF-1 were washed with PBS (Gibco, USA) and recovered through trypsin treatment. The recovered cells were washed with PBS (Gibco, USA), fixed with 2% paraformaldehyde (molecular probes) for 20 min, and permeabilized with 0.02% Triton-X (Gibco, USA) for 15 min. The cells were primarily incubated with the rabbit polyclonal anti-ovalbumin antibody (1:1,000 [1 mg/mL] dilution, Abcam, UK) for 30 min at room temperature and then secondarily incubated with the mouse anti-rabbit FITC antibody (1:1,000 [2 mg/mL] dilution, Abcam, UK) for 20 min at room temperature, only the secondary antibody staining was used as an isotype control. The two abovementioned antibodies were diluted with permeabilization buffer (Gibco, USA). The cells were subsequently washed three times and each 1×10^4^ cells were analyzed using a FACSCalibur (BD Bioscience, Franklin Lakes, NJ, USA).

### Vector construction and transfection

To validate the ovalbumin promoter activity into cOECs, the mutated 4.4-kb ovalbumin gene promoter (Mut-4.4-kb-pOV) with a 1-kb deletion between the EREs and the 2.8-kb ovalbumin promoter was constructed [10]. This DNA fragment was entirely synthesized, and then cloned into pGL4.11 (luc2p) reporter vector (Promega, Madison, WA, USA). cOECs at passage 4 and DF-1 were transfected with Lipofectamine 3000 (Invitrogen, USA) according to the manufacturer’s instructions. Briefly, cOECs and DF-1 were seeded into a 12-well cell culture plate, and the transfection was performed at 70% cell density, based on molecular ratio of each vector, namely, mock/pGL4.11 and Mut-4.4-kb-pOV/pGL4.11. For transfection, 1.5 μL of Lipofectamine 3000 reagent suspended in 50 μL of Opti-MEM (Gibco, USA) and 1 μg of each pGL4.11 firefly DNA, 0.01 μg of pGL4.74 (hRluc) renilla DNA (Promega, USA), and 2 μL of p3000 reagent suspended in 50 μL of Opti-MEM were mixed for 20 min at room temperature. The mixture of 100 μL per well was added to each well of the 12-well cell culture plate.

### Luciferase reporter analysis

For the reporter analysis, the cell culture medium was removed, and 250 μL of passive lysis buffer (Promega, USA), was added to each well of the 12-well cell culture plate. The plate was then stirred for 20 min for cell lysis, and the lysed cells were transferred to a 1.5 mL Eppendorf tube and centrifuged at 12,000 rpm for 10 min at room temperature. Each lysate of 20 μL was transferred to a 96-well white micro plate (Nunc, Rochester, NY, USA), and the luciferase activities were measured using a Centro LB 960 luminometer (Berthold, Bad Wildbad, Germany). The luciferase ratio for each vector was calculated as follows: (firefly luminescence)/(Renilla luminescence), and the relative luciferase ratio was calculated as follows: (mock/pGL4.11 luciferase ratio)/(Mut-4.4-kb-pOV/pGL4.11 luciferase ratio).

### Statistical analysis

The statistical analyses were conducted using GraphPad Prism statistical software (GraphPad Prism 5.03. software). Student’s t-test was used for the comparison of the relative luciferase ratio of Mut-4.4-kb-pOV/pGL4.11 and mock/pGL4.11, and p-values less than 0.05 was considered to indicate statistical significance. Error bars denote the standard error of the mean.

## RESULTS

### Isolation and *in vitro* culture of chicken oviduct epithelial cells

**Figure 1:**
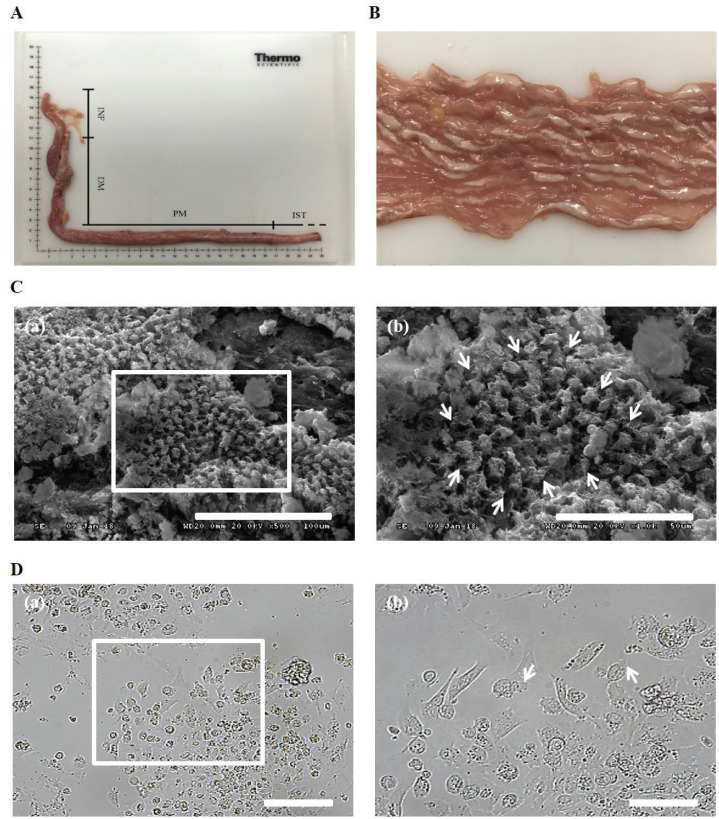
Structure analysis of the inner surface of isolated chicken oviduct tissue. (A) Total length of the chicken oviduct from the infundibulum to the edge of the magnum section. INF, infundibulum; DM, distal magnum; PM, proximal magnum; IST, isthmus. (B) Inner surface of chicken oviduct magnum. (C) Scanning electron microscopy (SEM) images of the magnum epithelium layer of chicken oviduct tissue. Scale bar: 100 μm (a) and 50 μm (b). The white arrows indicate the portion of ciliated surface. (D) Verification of the cilia of isolated oviduct cells. Scale bars: 200 μm (a) and 100 μm (b). The white arrows indicate cilia of the nonsecretory cells with a cobblestone shape.

**Figure 2:**
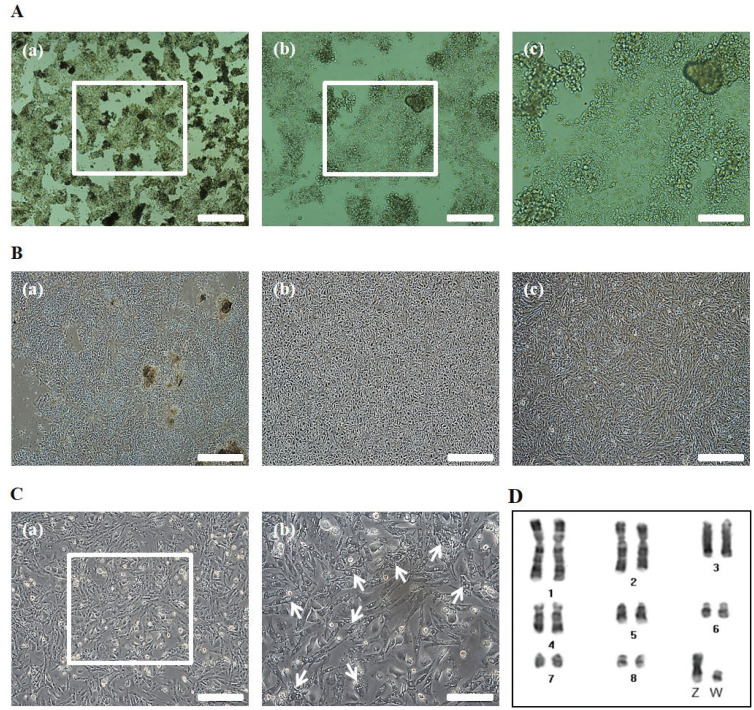
Morphological analysis of the isolated chicken oviduct cells. (A) Isolated chicken oviduct cells at passage 0, Scale bars: 500 μm (a), 200 μm (b), and 100 μm (c). (B) Morphological change of the cell population at passage 0 (a), 1 (b), and 2 (c). Scale bars: 500 μm. (C) Fibroblast-like cells with secretory granules at passage 2. Scale bars: 200 μm (a) and 100 μm (b). The white arrows indicate the portion of fibroblast-like cells with secretory granules. (D) Chromosomal karyotyping of cultured chicken oviduct epithelial cells. The chromosomes for sex chromosomes (Z and W) and macrochromosomes (chromosomes 1 to 8) were banded by Giemsa staining.

To obtain cOECs, we isolated oviduct tissue from egg-laying WL hens and analyzed the structure of the inner surface. The length of oviduct tissue separated from the infundibulum to the proximal magnum was approximately 36 cm (Figure 1A), and the inner surface of the magnum epithelium of the chicken oviduct tissue showed thick, anastomosing folds of the mucosa (Figure 1B). The magnum portion of a chicken oviduct tissue was subsequently separated and analyzed by SEM. As shown in Figure 1C, the magnum showed ciliated surface, and ciliated cells were observed in the culture plate after isolating the cells from oviduct tissue (Figure 1D). Through our isolation method, almost of the primary cOECs at passage 0 composed of colony-initiating cells (Figure 2A). The population of cOECs at passage 1 was heterogeneously composed of epithelial-like ciliated nonsecretory cells with a cobble stone shape and fibroblast-like cells (Figure 2B). From at passage 2, the population of epithelial-like ciliated nonsecretory cells continuously diminished, whereas the fibroblast-like cells were maintained at least fifth passages, and most of these cells showed secretory granules (Figure 2C). Furthermore, chromosomal karyotyping of the cultured cOECs showed normal chicken chromosome (2n = 78) (Figure 2D). Altogether, these results demonstrate the efficient isolation and short-term *in vitro* cultivation of the oviduct epithelial cells.

### Characterization of the *in vitro* cultured chicken oviduct epithelial cells

**Figure 3:**
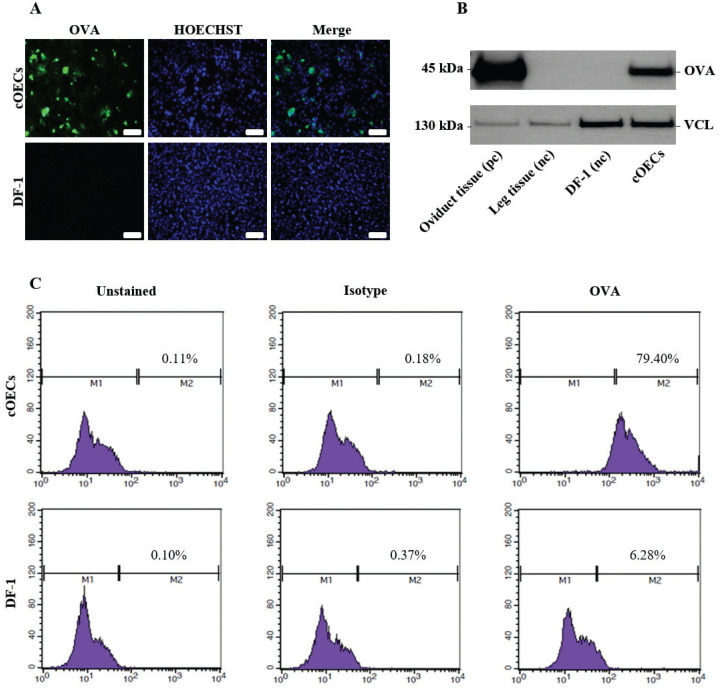
Characterization of the isolated chicken oviduct epithelial cells. (A) Ovalbumin (OVA) protein expression in the oviduct epithelial cells was detected by immunofluorescence analysis. After signal development, the cells were counterstained with Hoechst 33258. Scale bars: 100 μm. (B) Western blot analysis of oviduct epithelial cells. The results for chicken oviduct magnum as a positive control (pc), chicken leg muscle and DF-1 as a negative control (nc) are presented. The OVA and vinculin (VCL) proteins were used as oviduct-specific markers and loading controls. (C) Flow cytometry analysis of the percentage of ovalbumin positive cells in cOECs and DF-1. The cells were stained with an anti-OVA antibody, stained with isotype control antibody (Isotype) or not stained with any antibody (Unstained). The unstained and isotype groups were used as negative controls, and fibroblast cells (nc) stained with OVA antibody were also included in the analysis.

To characterize the cultured cOECs, we selected a set of chicken oviduct-specific markers. The cDNA from the oviduct magnum tissue, DF-1, and the cOECs were amplified by RT-PCR using chicken oviductal and epithelial cell markers (Table 1). The analysis of chicken oviduct-specific markers revealed that ovalbumin mRNA expression was clearly detected in magnum tissue and the cOECs. However, ESR1 mRNA, but not ovomucoid mRNA expression, was detected in all samples. The analysis with epithelial cell markers showed that occludin and E-cadherin mRNA expression were detected in the oviduct magnum tissue and cOECs, whereas cytokeratin 14 mRNA expression was detected in both DF-1 and cOECs but not in the oviduct magnum tissue. The RT-PCR results are summarized in Table 2. Based on the RT-PCR results, cOECs were qualitatively and quantitatively characterized through immunofluorescence, western blot, and flow cytometry analysis. As shown in Figure 3, the ovalbumin protein was clearly detected in the cOECs but not in DF-1 by immunofluorescence (Figure 3A). This ovalbumin protein was also clearly detected in the cOECs and the oviduct magnum tissue but not in leg muscle tissue and DF-1 by western blot analysis (Figure 3B). In flow cytometry analysis, ovalbumin protein expression in stained DF-1 was hardly detected (6.28%), in unstained DF-1 (0.10%), and in isotype control DF-1 (0.37%). In addition, only 0.11% and 0.18% were detected in unstained and in isotype control cOECs, whereas 79.40% of the stained cOECs showed positive detection of ovalbumin protein (Figure 3C). Taken together, the characterization results clearly demonstrate that ovalbumin protein, chicken oviduct-specific marker, was expressed in *in vitro* cultured cOECs.

### *In vitro* validation of the activity of the constructed chicken ovalbumin promoter

**Figure 4:**
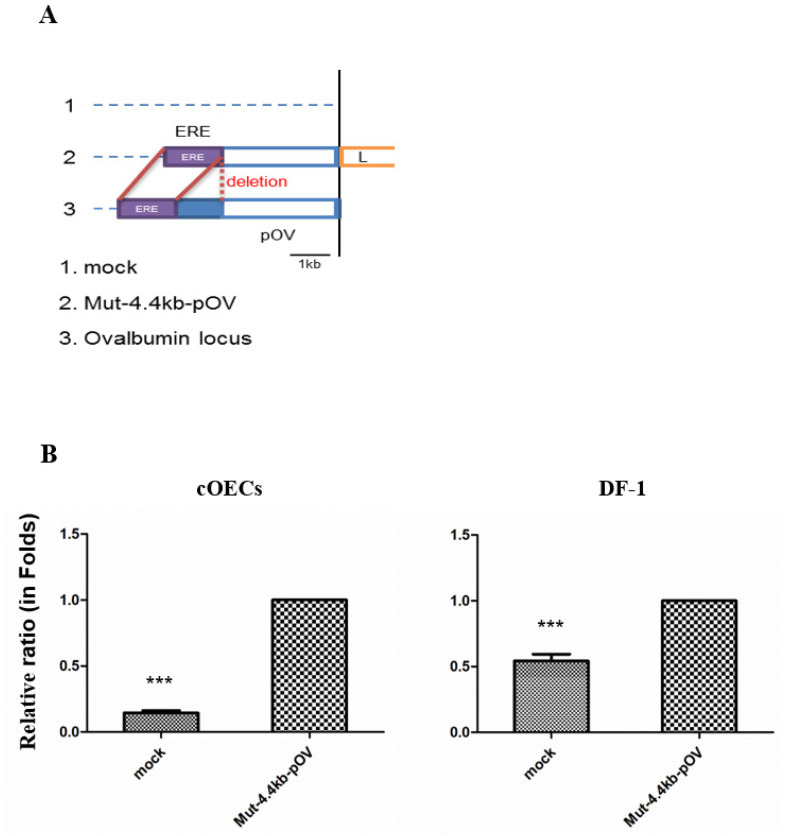
*In vitro* validation of the chicken ovalbumin promoter in the isolated chicken oviduct epithelial cells. (A) Diagram of the reporter vector cloned with the mutated 4.4-kb ovalbumin promoter with a 1-kb deletion between the estrogen response elements (EREs) region and the 2.8-kb promoter. (B) Relative luciferase ratio in chicken fibroblast cells and isolated chicken oviduct epithelial cells (cOECs). The data are presented relative to the values obtained for the Mut-4.4-kb-pOV group. The luciferase ratio for each vector was calculated as follows: (firefly luminescence)/(Renilla luminescence), and the relative luciferase ratio was calculated as follows: (mock/pGL4.11 luciferase ratio)/(Mut-4.4-kb-pOV/pGL4.11 luciferase ratio). The error bars indicate the means±standard error of the mean (n = 3). *** p<0.001 compared with the control (Mut-4.4-kb-pOV group).

To validate the activity of the ovalbumin promoter in the cOECs, we constructed a reporter vector cloned with a 4.4-kb ovalbumin promoter with an approximately 1-kb deletion between the EREs and the 2.8-kb ovalbumin promoter, and this vector was denoted Mut-4.4-kb-pOV/pGL4.11 (Figure 4A). The Mut-4.4-kb-pOV/pGL4.11 and mock/pGL4.11 vectors were transfected into the cOECs and DF-1, respectively, and the ovalbumin promoter activity in each cell was analyzed through a luciferase assay. The comparative luciferase analysis results showed that Mut-4.4-kb-pOV/pGL4.11 resulted in a 7.1-fold (p<0.001) higher relative luciferase ratio in the cOECs than the mock/pGL4.11 vector. However, the variation in the relative luciferase ratio between the Mut-4.4-kb-pOV/pGL4.11 and mock/pGL4.11 vectors was decreased by 2.0-fold (p<0.001) in DF-1 (Figure 4B). We thus verified that the constructed ovalbumin promoter exhibited specific promoter activity in the cultured cOECs. Collectively, these results demonstrate the successful preparation of cOECs from the area between the infundibulum and magnum in chicken oviduct tissue, the characterization of the cOECs using ovalbumin as a marker of the chicken oviduct, and the analysis of the expression and regulation of specific genes by the ovalbumin promoter in these cells.

## DISCUSSION

In the present study, we established a protocol for the isolation and characterization of cOECs and showed that these cOECs can be used for the evaluation of chicken ovalbumin promoter activity. Many transgenic birds have been generated in recent years, and previous studies have shown that lentivirus-mediated transgenesis achieves a high level of productivity in chickens [2] and quail [16].

Cloning of the lentivirus vector with chicken ovalbumin promoter allows efficient expression of a transgene in the chicken oviduct, leading to the development of ovalbumin promoters of different size, and the expression level of the transgene depends on the type of ovalbumin promoters. The 1.35-to-2.8-kb ovalbumin promoter consisting of steroid-dependent response elements and negative response elements induces tissue-specific expression, but the resulting expression level is considered low for industrial use [17]. However, insertion of the 673 or 675 bp of EREs in front of the 5′-flanking region of the 2.8-kb ovalbumin promoter results in significantly enhanced expression level of the transgene [5,8]. Based on these findings, it was then necessary to evaluate the developed ovalbumin promoters *in vitro* prior to the generation of transgenic chickens, and thus, the establishment of a protocol for cOECs is important, even though the culturing of cOECs is in challenging [18,19].

cOECs differentiate into ciliated nonsecretory cells, secretory cells, and tubular gland cells [13,20]. As indicated by our results, the isolated oviduct cells at passage 0 were populated by cells with similar morphological shapes, which are denoted colony-initiating cells and also considered progenitor cells. At passage 1, the population heterogeneously consisted of epithelial-like ciliated nonsecretory cells with a cobblestone shape and fibroblast-like cells. Starting at passage 2, the number of epithelial-like ciliated cells decreased until these cells were hardly observed under a microscope, but the fibroblast-like cells were stably maintained at least until passage 5. We also observed secretory granules in these fibroblast-like cells, which demonstrates that the cells are likely mature tubular gland cells [21].

An optimal method for the isolation of cOECs has been reported, and the results indicate that epithelial-like cells were retained from the infundibulum of the chicken oviduct in keratinocyte medium [14]. The different morphologies of the oviduct cells isolated from the infundibulum, distal magnum, and proximal magnum were observed, and these cells populations consisted of colony-initiating cells, epithelial cells showing cobblestone shape, and fibroblast-like cells, respectively [15]. In our preliminary experiments, we attempted to culture the isolated oviduct cells in DMEM supplemented with FBS, but the resulting cell populations consisted exclusively of fibroblast cells did not contain secretory granules, which differ from the cell populations obtained in keratinocyte medium. Additionally, we isolated the oviduct cells, not dividing into each infundibulum, distal magnum, and proximal magnum, and the morphological changes in the cells populations were observed mostly according to cells passages.

After culturing, we investigated suitable two types of markers for characterizing the cOECs. Among three oviductal and three epithelial markers, the primer pairs for ESR1 and E-cadherin were firstly designed in this study, but the primers used for ovomucoid, ovalbumin, occludin, cytokeratin 14, and β-actin were based on those designed in previous studies [15,22,23]. According to our RT-PCR results, the ovalbumin, occludin, and E-cadherin proteins can be used as oviductal and epithelial markers, and subsequent immunofluorescence, western blot, and flow cytometry analysis, clearly demonstrated that the cOECs were cells that expressed ovalbumin protein.

We finally demonstrated that the mutated 4.4-kb chicken ovalbumin promoter exhibited 7.1-fold higher activity than the negative control. Based on these results, we will utilize the cOECs for *in vitro* validation of the chicken ovalbumin promoter. In summary, this study aimed to isolate and characterize cOECs and to investigate the activity of the ovalbumin promoter in these cells. The results revealed that the oviduct cells expressing the ovalbumin protein could be successfully isolated using our method, and their characterization clearly showed that the cells exhibited an ovalbumin marker. These results can strongly aid the development of advanced chicken promoter for the production of recombinant proteins and ultimately the generation of transgenic hens.

## Figures and Tables

**Table 1:** Primer sequences for reverse transcription polymerase chain reaction amplification of the specific genes

Gene	Primer sequences	Size (bp)	References
Ovalbumin	F: CGTTCAGCCTTGCCAGTAGA R: AGTATTCTGGCAGGATTGGGT	60	Stadnicka et al [15]
Ovomucoid	F: TATGCCAACACGACAAGCGA R: CCCCCTGCTCTACTTTGTGG	133	Stadnicka et al [15]
Estrogen receptor 1	F: ACCACTATGGGGTCTGGTCT R: TCTGCGGTCTTTCCGGATTC	197	this study
Occludin	F: GAGGAGTGGGTGAAGAACGTG R: GGTGCCCGAGGGGTAGTA	150	Stadnicka et al [15]
Cytokeratin 14	F: GCGAGGACGCCCACATCTCTTC R: TGAGCGCCATCTGCTCACGG	150	Couteaudier et al [23]
E-cadherin	F: TGGATGGTGCCTTCAGCATT R: GATAGGGGGCACGAAGACAG	215	this study
Beta actin	F: CACAGATCATGTTTGAGACCTT R: CATCACAATACCAGTGGTACG	101	Sevane et al [22]

F, forward primer; R, reverse primer.

**Table 2:** mRNA expression of the specific genes in oviduct magnum, DF-1, and the cOECs

Type	Genes	Magnum	DF-1	cOECs
Oviduct marker	Ovalbumin	+	ND	+
	Ovomucoid	ND	ND	ND
	Estrogen receptor 1	+	+	+
Epithelial marker	Occludin	+	ND	+
	Cytokeratin 14	ND	+	+
	E-cadherin	+	ND	+
Control	Beta actin	+	+	+

cOECs, chicken oviduct epithelial cells; ND, not determined.
